# Independent association between subjective cognitive decline and frailty in the elderly

**DOI:** 10.1371/journal.pone.0201351

**Published:** 2018-08-02

**Authors:** Tsung-Jen Hsieh, Hsing-Yi Chang, I-Chien Wu, Chu-Chih Chen, Hui-Ju Tsai, Yen-Feng Chiu, Shu-Chun Chuang, Chao A. Hsiung, Chih-Cheng Hsu

**Affiliations:** 1 Institute of Population Health Sciences, National Health Research Institutes, Miaoli County, Taiwan; 2 Department of Health Services Administration, China Medical University, Taichung City, Taiwan; 3 Department of Family Medicine, Min-Sheng General Hospital, Taoyuan, Taiwan; Nathan S Kline Institute, UNITED STATES

## Abstract

**Background:**

The relationship between subjective cognitive decline and frailty, two components of the so-called reversible cognitive frailty, in the elderly remains unclear. This study aims to elucidate whether this association exists, independent of confounding factors such as nutritional status, kidney function, inflammation, and insulin resistance.

**Methods:**

2386 participants (≥ 65 years of age) selected from the Healthy Aging Longitudinal Study in Taiwan (HALST) study. Fried frailty phenotype was adopted to quantify frailty status. We classified cognitive status into two categories—subjective cognitive decline (SCD), and normal cognition—and used polytomous logistic regressions to investigate the associations between SCD and frailty.

**Results:**

There were 188 (7.88%), 1228 (51.47%), and 970 (40.65%) participants with frailty, pre-frailty, and robustness, respectively. Compared to those with normal cognition, elders with SCD were more likely to have pre-frailty (odds ratio [OR]: 1.36, 95% confidence interval [CI]: 1.10–1.67, *p* = 0.004) or frailty (OR: 1.78, 95% CI: 1.23–2.58, *p* = 0.002) after adjusting for age, gender, education level, comorbidity, nutritional status, kidney function, and biochemical-related factors.

**Conclusions:**

A significant association between subjective cognitive decline and frailty was revealed in this study. Subjective cognitive decline was positively associated with pre-frailty or frailty even after adjusting for potential confounding factors. Our results can provide useful references in understanding mechanisms and developing suitable preventive strategies for the elderly with reversible cognitive frailty.

## Introduction

Frailty and cognitive function impairment are two perilous clinical conditions for the elderly. Frailty in the elderly is a complicated and multifactorial syndrome that could result from the interplay of physiological, genetic, and environmental factors [1]. In the past decade, it has received much medical attention because the frail state can increase vulnerability and risks of adverse health events, such as falls, disability, hospitalization, and mortality [2, 3]. Therefore, early detection and prevention of frailty is of crucial importance for both researchers and clinicians. A number of cross-sectional and longitudinal studies have shown that frailty and cognitive impairment may share similar etiologies. For example, progression of frailty is associated with incident Alzheimer's disease (AD) and an accelerated rate of cognitive decline in the elderly [4, 5]. On the other hand, subjects with poor cognitive performance or with cognitive impairment are independently associated with increased risk of frailty [6, 7]. A study has shown that cognitive decline is associated with longitudinal frailty state transitions among elders with mild cognitive impairment (MCI), and with mild or moderate AD [8].

Subjective cognitive decline (SCD) is a self-recognized decline in cognitive performance in comparison to previous experience [9]. The results of previous studies have reported that SCD is associated with poorer cognitive function for the elderly [10, 11]. In longitudinal studies, it is also a clinical risk factor for future cognitive decline [12], and a first clinical manifestation of AD [13].

In recent years, an international consensus group has recognized cognitive frailty as a heterogeneous clinical symptom of simultaneous presence of both physical frailty and cognitive impairment in the absence of dementia [14]. Moreover, two subtypes of cognitive frailty have been proposed to refine the definition and potential mechanisms of this clinical manifestation based on the severity of cognitive impairment: reversible cognitive frailty and potentially reversible cognitive frailty [15]. The former is indicated by presence of both SCD and physical frailty, and the latter is defined as presence of both MCI and physical frailty. Recently, there is supportive evidence that people with (reversible) cognitive frailty have an increased risk for adverse health outcomes (e.g., functional disability and impaired quality of life), overall dementia, and all-cause mortality [16, 17]. Therefore, in a perspective of primary risk prevention, it is worth to clarify the link of this pre-MCI indicator (SCD) to frailty phenotype in the elderly. However, to our knowledge, the relationship between subjective cognitive decline and frailty in the elderly remains unclear. In addition, many factors—including systemic inflammation, nutritional status, kidney function, and insulin resistance—are capable of playing a vital role in the process of frailty development [18–21].Therefore, the objectives of this study were to elucidate the association between subjective cognitive decline and frailty in the elderly and to examine whether this association is independent of factors related to nutritional status, kidney function, inflammation, and insulin resistance.

## Methods

### Participants

**Fig 1:**
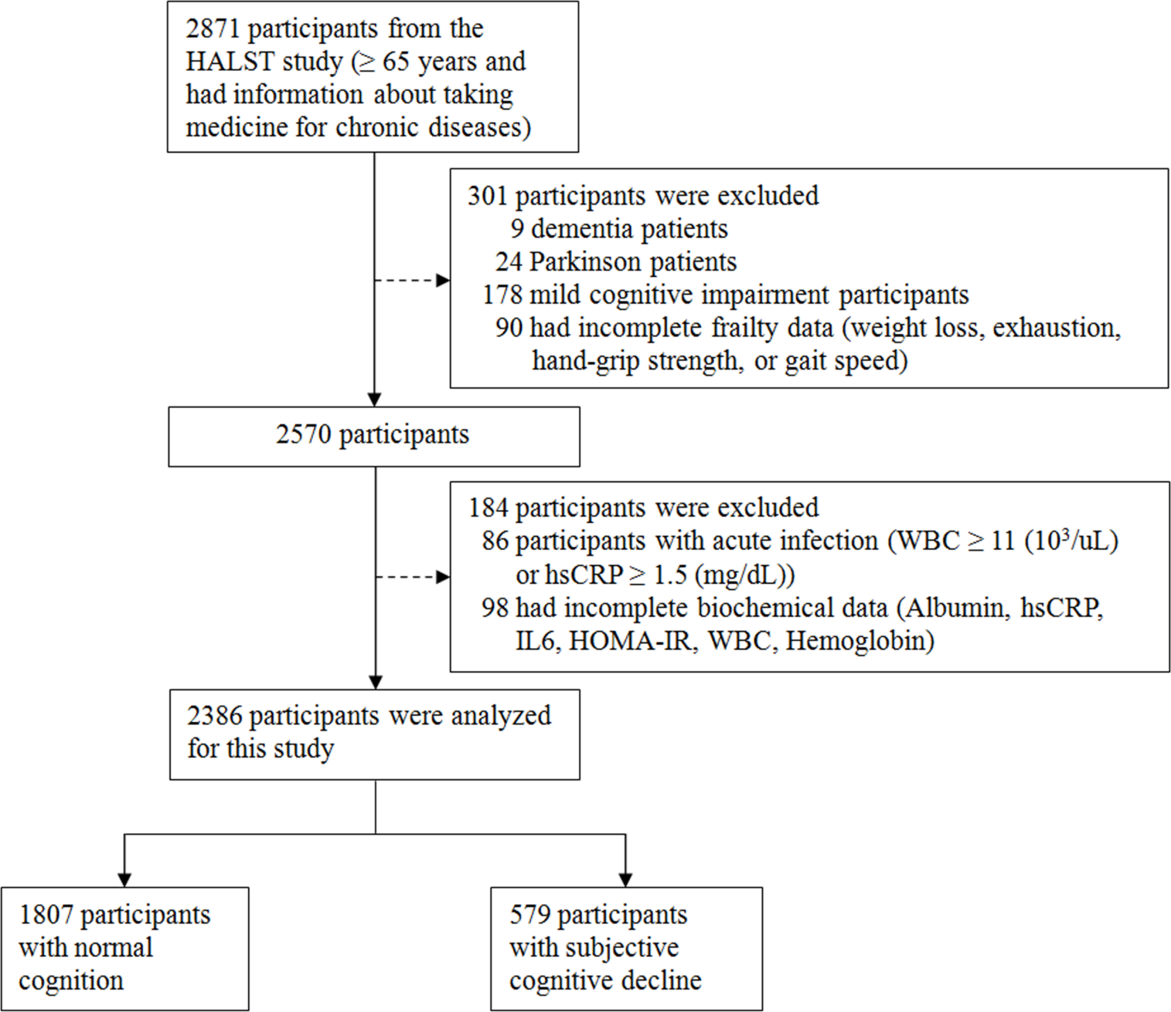
Flow chart of the selection of subjects from the HALST study.

All study participants were selected from the Healthy Aging Longitudinal Study in Taiwan (HALST), a longitudinal ageing cohort study recruiting community dwellers aged 55 and older during 2009–2013. A detailed study design of the HALST has been provided elsewhere [22]. From the HALST, we selected those who were 65 years of age or older and had information about taking medicine for chronic diseases as the study subjects for further analysis. Following the international consensus [9], people with SCD must have normal cognitive performance. Therefore, participants with dementia, Parkinsonism (tend to have impaired cognitive function), or MCI were excluded from analysis. Elderly were defined as having MCI if the score of the Mini-Mental State Examination (MMSE) [23] < 17 for illiterate subjects, or < 20 for subjects with education of 1–6 years, or < 24 for subjects with education > 6 years [24]. In addition, the physician-diagnosed dementia and Parkinsonism were identified during interview. The study subjects’ selection flow was depicted in Fig 1 (*n* = 2386). Data were collected through home interviews, clinical examinations, and laboratory analysis. The study protocol of the HALST was approved by the ethics committee of the National Health Research Institutes in Taiwan (protocol number: EC0970608); and all study participants gave written informed consent independently.

### Assessment of frailty and subjective cognitive decline

The frailty phenotype proposed by Fried [2] was adopted to quantify the frailty status of study participants. The criteria have five aspects: unintentional weight loss, exhaustion, poor muscle strength, slowness, and low physical activity. Unintentional weight loss was defined as involuntary loss of more than 3 kg of body weight during the previous year. Exhaustion was assessed by responses to the following two statements from the Center for Epidemiologic Studies Depression (CES-D) scale during the past week: “I felt that everything I did was an effort” and “I could not get going.” [25]. If participants responded “occasionally (3–4 days)” or “mostly (5–7 days)” to either of these two statements, then they would be categorized as positive for the aspect of exhaustion. Poor muscle strength and slowness were respectively defined by low hand-grip strength and slow gait speed, which were correspondingly determined by the gender- and body mass index-specific cutoff points of hand-grip strength, as well as the gender- and height-specific cutoff points of gait speed obtained from the previous study [26]. Moreover, physical activity was evaluated by the kilocalorie energy expenditure [27] and the following two questions: “Did you do any regular exercise during the past year?” and “Did you do any strenuous activity during the past year?” Participants having low calorie consumption (< 685 kcal/week for male, and < 420 kcal/week for female) [28] or answering “no” to both these questions were defined as low physical activity.

People who had three or more of Fried’s five criteria were identified as frail; those meeting one or two criteria were recognized as pre-frail; and those with none of the Fried criteria were categorized as robust.

The SCD Initiative (SCD-I) Working Group has identified 34 self-report measures comprising 640 cognitive self-report items from 19 studies [29]. However, until recently there was no single accepted approach for evaluation of SCD. An optimal approach about assessment of pre-clinical cognitive impairment for the elderly better involve events that they encounter frequently in their daily lives (e.g., medical history), as suggested by the SCD-I Working Group [29]. Thus, following the concept of SCD proposed by the SCD-I Working Group [9], in this study we defined participants with SCD if the subjects had a normal cognitive function test by MMSE but self-reported that they often forgot to take their regular drugs during the past year. Those without SCD were recognized as having normal cognition.

### Other medical and laboratory assessments

The past history of chronic and cardiovascular diseases—including angina, myocardial infarction, stroke, cancer, and chronic respiratory disease (CRD)—was identified by self-reported conditions that were informed by a physician. Hypertension was defined as having a baseline systolic blood pressure ≥ 140 mmHg, a baseline diastolic blood pressure ≥ 90 mmHg, or by self-report. Diabetes mellitus (DM) was defined as having a baseline fasting blood glucose ≥ 126 mg/dL, having a baseline HbA_1C_ ≥ 6.5%, or by self-report. As defined by the International Diabetes Federation [30], metabolic syndrome (MetS) is the presence of central obesity plus any two of four additional factors: raised triglycerides, blood pressure, fasting plasma glucose, or reduced high-density lipoprotein-cholesterol. The Chinese version of the Barthel index [31] and Lawton and Brody’s measurement [32] were utilized to assess the activities of daily living (ADL) and instrumental activities of daily living (IADL), respectively. Poor nutritional status was defined as having a serum albumin < 4.0 g/dL [33]. Kidney function was categorized as low, moderate, high, or very high risk of prognosis of chronic kidney disease (CKD), according to the Kidney Disease Improving Global Outcomes (KDIGO) clinical practice guideline [34]. Moreover, clinical biochemical-related factors were measured by high sensitive c-reactive protein (hsCRP), interleukin-6 (IL-6), white blood cell (WBC), hemoglobin, and insulin-resistance assessed by the homeostasis model assessment of insulin resistance (HOMA-IR) [35].

### Statistical analysis

Continuous variables were expressed as mean ± standard deviation (SD); and categorical variables were expressed as number (percentage). Differences in demographic data, past medical history, cognitive function test, functional ability, physical performance, and frailty characteristics between the two investigated groups were tested by utilizing the nonparametric Wilcoxon rank-sum test for non-normally distributed continuous variables (Shapiro-Wilk test, *p* < 0.05) and the chi-square test or Fisher's exact test for categorical variables. Crude association among frailty status (robust, pre-frailty, and frailty), cognitive status, nutritional status, kidney function, and clinical biochemical-related factors were analyzed by chi-square test. Moreover, a series of polytomous logistic regressions were employed to investigate an adjusted association between SCD and frailty by adjustment for age, gender, education level, past medical history (hypertension, stroke, DM, and Mets), nutritional status, kidney function, inflammation (hsCRP, IL-6, and WBC), insulin resistance, and hemoglobin, since these variables were associated with SCD or frailty. The regression models were started with the uncorrected covariate (only included SCD in the model) and then sequentially added each confounding factor and the interaction term between SCD and adjusted factor to explore the separate contribution to frailty. A correlation matrix among these adjusted variables were also calculated to clarify the relationships of these potential confounding factors. In addition, adjusted association between SCD and each frailty characteristic was evaluated. All data analyses were carried out using SAS version 9.4 statistical software (SAS Institute Inc. Cary, NC, USA). The statistical significance of all tests was evaluated at a predetermined significance level of 0.05.

## Results

A total of 2386 participants—comprising 579 SCD, and 1807 normal cognition—were selected from the HALST study for further analysis. Characteristics of participants by SCD or normal cognition are shown in Table 1. The mean age of the participants was 73.23 ± 5.71 years, and 51.26% were women. There was an increasing difference, for subjects with SCD, in the prevalence of past medical history (hypertension, DM, and MetS) and frailty characteristics (unintentional weight loss, exhaustion, poor muscle strength, slowness, and low physical activity), compared to those with normal cognition. Table 2 shows there were 188 (7.88%), 1228 (51.47%), and 970 (40.65%) participants with frailty, pre-frailty, and robustness, respectively. The unadjusted results show the percentages of SCD differed significantly in robust, pre-frailty and frailty subjects. Compared to their robust counterparts, the pre-frail subjects had a higher percentage of poor nutritional status, more advanced CKD, and worse biochemical-related factors, with the frail subjects showing even worse conditions.

**Table 1:** Characteristics of participants between SCD and normal cognition.

Characteristics	All (*n* = 2386)	Normal cognition (*n* = 1807)	SCD (*n* = 579)	*p*-value
Age (years)	73.23 ± 5.71	73.27 ± 5.79	73.11 ± 5.47	0.939
Gender (women)	1223 (51.26)	879 (48.64)	344 (59.41)	< 0.001
Education level (> 6 years)	1010 (42.33)	809 (44.77)	201 (34.72)	< 0.001
Past medical history				
Hypertension	1435 (60.14)	1054 (58.33)	381 (65.80)	0.001
Angina	46 (1.93)	35 (1.94)	11 (1.90)	0.955
MI	45 (1.89)	31 (1.72)	14 (2.42)	0.280
Stroke	137 (5.74)	102 (5.64)	35 (6.04)	0.719
DM	724 (30.34)	516 (28.56)	208 (35.92)	< 0.001
Cancer	160 (6.71)	121 (6.70)	39 (6.74)	0.974
CRD	70 (2.93)	55 (3.04)	15 (2.59)	0.574
MetS	833 (34.91)	589 (32.60)	244 (42.14)	< 0.001
Cognitive function test				
MMSE (points)	26.33 ± 3.14	26.45 ± 3.07	25.94 ± 3.31	0.002
Functional ability				
ADL (> 60 points)	2384 (99.92)	1805 (99.89)	579 (100.00)	1.000
IADL (> 6 points)	2349 (98.45)	1785 (98.78)	564 (97.41)	0.020
Physical performance				
Hand-grip strength (kg)	26.26 ± 9.30	26.77 ± 9.34	24.68 ± 9.02	< 0.001
Gait speed (m/s)	0.84 ± 0.25	0.85 ± 0.25	0.81 ± 0.26	< 0.001
Frailty characteristics				
Unintentional weight loss	180 (7.54)	118 (6.53)	62 (10.71)	< 0.001
Exhaustion	155 (6.50)	107 (5.92)	48 (8.29)	0.044
Poor muscle strength	470 (19.70)	388 (18.71)	132 (22.80)	0.031
Slowness	543 (22.76)	386 (21.36)	157 (27.12)	0.004
Low physical activity	877 (36.76)	639 (35.36)	238 (41.11)	0.013

**Abbreviation:** SCD, Subjective Cognitive Decline; MI, Myocardial Infarction; DM, Diabetes Mellitus; CRD, Chronic Respiratory Disease; MetS, Metabolic Syndrome; MMSE, Mini-Mental State Examination; ADL, Activities of Daily Living; IADL, Instrumental Activities of Daily Living.

Note: Data are expressed as means ± SD or *n* (%).

**Table 2:** Characteristics of participants in different frail conditions.

Variables	Robust (*n* = 970)	Pre-frailty (*n* = 1228)	Frailty (*n* = 188)	*p*-value
Age (≥ 75 years)	229 (23.61)	444 (36.16)	110 (58.51)	< 0.001
Gender (women)	485 (50.00)	641 (52.20)	97 (51.60)	0.589
Education level (> 6 years)	477 (49.18)	487 (39.66)	46 (24.47)	< 0.001
SCD	195 (20.10)	323 (26.30)	61 (32.45)	< 0.001
Cognitive function test				
MMSE (points)	27.03 ± 2.75	26.06 ± 3.20	24.51 ± 3.61	< 0.001
Past medical history				
Hypertension	528 (54.43)	778 (63.36)	129 (68.62)	< 0.001
Angina	11 (1.13)	31 (2.52)	4 (2.13)	0.061
MI	17 (1.75)	26 (2.12)	2 (1.06)	0.567
Stroke	33 (3.40)	80 (6.51)	24 (12.77)	< 0.001
DM	251 (25.88)	390 (31.76)	83 (44.15)	< 0.001
Cancer	62 (6.39)	84 (6.84)	14 (7.45)	0.838
CRD	28 (2.89)	35 (2.85)	7 (3.72)	0.799
MetS	303 (31.24)	446 (36.32)	84 (44.68)	< 0.001
Poor nutritional status (Alb < 4.0 g/dL)	15 (1.55)	53 (4.32)	16 (8.51)	< 0.001
Prognosis risks of CKD				< 0.001
Low	702 (72.37)	750 (61.07)	81 (43.09)	
Moderate	174 (17.94)	307 (25.00)	46 (24.47)	
High	63 (6.49)	87 (7.08)	34 (18.09)	
Very high	31 (3.20)	84 (6.84)	27 (14.36)	
Biochemical factors				
hsCRP ≥ 0.19^a^ (mg/dL)	205 (21.13)	345 (28.09)	59 (31.38)	< 0.001
IL-6 ≥ 2.26^a^ (pg/mL)	176 (18.14)	333 (27.12)	87 (46.28)	< 0.001
HOMA-IR ≥ 2.35^a^	203 (20.93)	332 (27.04)	63 (33.51)	< 0.001
WBC ≥ 6.60^a^ (10^3^/uL)	218 (22.47)	348 (28.34)	68 (36.17)	< 0.001
Hemoglobin ≤ 12.0 (g/dL)	91 (9.38)	163 (13.27)	45 (23.94)	< 0.001

**Abbreviation:** SCD, Subjective Cognitive Decline; MMSE, Mini-Mental State Examination; MI, Myocardial Infarction; DM, Diabetes Mellitus; CRD, Chronic Respiratory Disease; MetS, Metabolic Syndrome; Alb, Albumin; CKD, Chronic Kidney Disease; hsCRP, High Sensitive C-Reactive Protein; IL-6, Interleukin-6; HOMA-IR, Homeostasis Model Assessment of Insulin Resistance; WBC, White Blood Cell.

Note: Data are expressed as means ± SD or *n* (%).

^a^ 75th percentile.

Supplementary S1 Table (in the supporting information) shows a correlation matrix of adjusted confounding factors in regression models, and most of factors revealed small correlations to other variables. For simplicity, partial results of polytomous logistic regression models are summarized in Table 3. As shown in Table 3, compared to those with normal cognition, elders with SCD were more likely to have pre-frailty (odds ratio [OR]: 1.36, 95% confidence interval [CI]: 1.10–1.67, *p* = 0.004) or frailty (OR: 1.78, 95% CI: 1.23–2.58, *p* = 0.002) after adjusting for age, gender, education level, major past medical history, and other confounding factors such as nutritional status, kidney function, and biochemical-related factors (Model 3 in Table 3). The complete results of regression models adjusted sequentially by each cofounding factor are available in supplementary S2 Table. There were not interaction effects between SCD and each added-covariate.

**Table 3:** Adjusted odds ratios of SCD versus normal cognition for the elderly with pre-frailty or frailty (robust as reference category).

Models	SCD versus normal cognition for pre-frailty OR (95% CI)	*p*-value	SCD versus normal cognition for frailty OR (95% CI)	*p*-value
Model 1^a^	1.41 (1.15–1.73)	0.001	1.95 (1.36–2.78)	< 0.001
Model 2^b^	1.36 (1.11–1.67)	0.004	1.82 (1.27–2.61)	0.001
Model 3^c^	1.36 (1.10–1.67)	0.004	1.78 (1.23–2.58)	0.002

**Abbreviation:** SCD, Subjective Cognitive Decline.

^a^ SCD and adjusted for age, gender, and education level.

^b^ SCD and adjusted for age, gender, education level, and past medical history of hypertension, stroke, diabetes mellitus, and metabolic syndrome.

^c^ SCD and adjusted for age, gender, education level, past medical history, nutritional status, kidney function, inflammation (hsCRP, IL-6, and WBC), insulin resistance, and hemoglobin.

As shown in Table 4, the association between SCD and frailty characteristics was revealed in some aspects (unintentional weight loss, slowness, and low physical activity).

**Table 4:** Adjusted odds ratios of SCD versus normal cognition for five frailty characteristics.

Frailty characteristics	SCD versus Normal cognition OR (95% CI)	*p*-value
Unintentional weight loss	1.67 (1.20–2.33)	0.003
Exhaustion	1.31 (0.91–1.89)	0.144
Poor muscle strength	1.23 (0.96–1.57)	0.095
Slowness	1.27 (1.01–1.61)	0.042
Low physical activity	1.26 (1.03–1.53)	0.023

**Abbreviation:** SCD, Subjective Cognitive Decline.

Note: The models were adjusted for age, gender, education level, past medical history (hypertension, stroke, diabetes mellitus, and metabolic syndrome), nutritional status, kidney function, inflammation (hsCRP, IL-6, and WBC), insulin resistance, and hemoglobin.

## Discussion

This study demonstrated that, compared to subjects with normal cognition, elders with SCD were more likely to be pre-frail or frail. In the full models controlled for many related confounding factors such as nutritional status, kidney function, inflammation, insulin resistance, and hemoglobin, this interrelationship between subjective cognitive decline and frailty remained unchanged.

Subjective cognitive decline in elderly is a common aging process. A study found that the prevalence of SCD ranged from 25% to 50% [36]. Recently, it is increasingly recognized that SCD is a clinical indicator of asymptomatic cognitive impairment and have a higher hazard of being MCI or dementia than those with normal cognition and free of SCD [37]. Several studies have investigated the relationship between cognitive impairment and frailty. For example, frail elderly people have a higher rate of cognitive impairment than those with pre-frailty or robustness [38]; additionally, significant relationships between frailty and MCI are revealed in the elderly [39], and frail elderly people are more likely to have cognitive decline and memory decline than robust ones [40]. However, the relationship between SCD and frailty remains unclear. The results of our study additionally found that elders with subjective cognitive decline were also more likely to be pre-frail or frail than those with normal cognition.

Recently, some studies have also pointed out that the severity of frailty is associated with poorer cognitive function in global cognition, verbal memory, processing speed, and working memory for depressed elders [41] and community-dwelling elders [42]. In our study, compared to subjects with robustness, those with pre-frailty or frailty also had poorer performances in global cognition. Moreover, frailty is associated with worse cognitive function in patients with incident hemodialysis [43]. Consistently, our results suggested that subjects with pre-frail or frail state were associated with more severe kidney function.

In general, it is known that age is one of the most important independent risk factors for cognitive impairment and dementia [44]; thus many age-associated processes that result in frailty among the elderly are also related to cognitive decline. A previous study has shown that poor muscle strength and slowness are most associated with the occurrence of MCI [4], which coincides with our finding that SCD is most associated with unintentional weight loss and slowness. A study has shown that gait speed was affected by episodic memory problems or executive function impairments [45]; thus, the association between frailty and SCD could be attributable to the interrelationship between slowness and poorer cognitive function in memory.

A recent study examined the independent and combined effects of inflammation and endocrine dysregulation on baseline frailty status and frailty progression among cognitively impaired community-dwelling elderly [20]. The results showed that elders with isolated pro-inflammatory state were 4.06 times more likely to be frail one year later. Other studies have also supported that chronic inflammation is implicated in the association between frailty and cognitive performance [46, 47]. Our results from polytomous logistic regression analyses showed that the effects of SCD on frailty was independent of factors related to inflammation (hsCRP, IL-6, and WBC). This provided an explicit clue that inflammation-related factors could not be a mediator in the relationship between SCD and frailty. Therefore, we hypothesized that there should be a common pathway to determine these two elderly syndromes (subjective cognitive decline and frailty). It is worth to do further study to investigate the biological mechanism behind this linkage.

People with cognitive frailty (cognitive impairment together with physical frailty) has recently been recognized as a target population for prevention of cognitive impairment, dementia, and disability [14, 15]. Our study revealed the well-known interrelationship between frailty and cognitive impairment may influence each other as early as at the SCD stage. Our results can provide useful references in understanding mechanisms and developing suitable strategies of secondary prevention of cognitive impairment and AD for the elderly with reversible cognitive frailty (SCD together with physical frailty), especially for those with positive AD biomarkers [48].

In this study, some limitations have to be acknowledged. First, the data analyzed in this study were derived from the first-wave survey of the HALST study. The cross-sectional study precluded us from elucidating causal relationships between SCD and frailty. Second, based on the suggestions of the SCD-I Working Group [29], we defined SCD by the specific question about forgetfulness to taking medicine regularly, which is the event that the elderly encounter frequently in their daily life, and the question about management of a medication schedule is an approach to assess cognitive function in organization domain for the elderly [49]. However, we recognize the SCD definition may be biased if a wealth of study subjects did not have to take regular medications. Fortunately, of the HALST participants, only 16% did not have to take regular drugs for their chronic conditions. When we excluded those subjects, the results did not change: the odds ratios of SCD versus normal cognition for the elderly with pre-frailty or frailty are 1.27 (95% CI: 1.03–1.58, *p* = 0.028) and 1.56 (95% CI: 1.07–2.28, *p* = 0.020), respectively. Third, the elderly with functional impairment (ADL < 60 or IADL < 6) could have poorer performance on cognitive function or physical measurement. Moreover, frailty was mostly related to cardiovascular diseases (e.g., stroke), which can cause vascular dementia and also has an impact on cognitive and physical functions. Nevertheless, when we excluded those subjects, the association between SCD and frailty remained unchanged (results not shown).

## Conclusions

In conclusion, the results from our study revealed a significant association between subjective cognitive decline and frailty in the elderly. Subjective cognitive decline was positively associated with pre-frailty or frailty even after adjusting for potential confounding factors. The results of this study indicate that further investigation of the intricate mechanisms between subjective cognitive decline and physical frailty and sequential development of preventive trials for this reversible cognitive frailty would be worthwhile.

## Supporting information

S1 Table Correlation matrix of adjusted confounding factors in polytomous logistic regression models. (PDF)

S2 Table Odds ratios of SCD versus normal cognition for the elderly with pre-frailty or frailty (robust as reference category) in unadjusted and adjusted models. (PDF)
