# Supplementary material for: Independent association between subjective cognitive decline and frailty in the elderly
Source: PLoS One. 2018 Aug 2;13(8):e0201351. doi: 10.1371/journal.pone.0201351 (PMC6072005; doi:10.1371/journal.pone.0201351)
Supplement: S1 Table — (PDF) [file pone.0201351.s001.pdf]

**Supporting information:** **Independent association between subjective cognitive decline and frailty in the elderly**

**S1 Table. Correlation matrix of adjusted confounding factors in polytomous logistic regression models.**

| factors | Gender | Education level | HTN | Stroke | DM | MetS | Poor nutritional status | Prognosis risks of CKD | hsCRP | IL-6 | WBC | HOMA-IR | Hemoglobin |
|---|---|---|---|---|---|---|---|---|---|---|---|---|---|
| **Age** | -0.110[a] | -0.076[a] | 0.161[a] | 0.036[a] | 0.003[a] | 0.029[a] | 0.108[a] | 0.258[b] | 0.080[b] | 0.232[b] | 0.050[b] | -0.036[b] | -0.156[b] |
| **Gender** | – | -0.237[c] | 0.018[c] | -0.069[c] | -0.022[c] | 0.181[c] | -0.028[c] | -0.101[a] | 0.015[a] | -0.078[a] | -0.104[a] | 0.048[a] | -0.409[a] |
| **Education level** | – | – | -0.032[c] | -0.018[c] | -0.016[c] | -0.087[c] | -0.030[c] | -0.066[a] | -0.113[a] | -0.060[a] | -0.085[a] | -0.018[a] | 0.100[a] |
| **HTN** | – | – | – | 0.109[c] | 0.135[c] | 0.264[c] | 0.021[c] | 0.222[a] | 0.102[a] | 0.094[a] | 0.139[a] | 0.127[a] | -0.031[a] |
| **Stroke** | – | – | – | – | 0.080[c] | 0.038[c] | 0.021[c] | 0.147[a] | 0.033[a] | 0.040[a] | 0.071[a] | 0.069[a] | 0.006[a] |
| **DM** | – | – | – | – | – | 0.234[c] | -0.017[c] | 0.234[a] | 0.061[a] | 0.061[a] | 0.210[a] | 0.282[a] | -0.071[a] |
| **MetS** | – | – | – | – | – | – | -0.011[c] | 0.186[a] | 0.147[a] | 0.112[a] | 0.156[a] | 0.257[a] | -0.016[a] |
| **Poor nutritional status** | – | – | – | – | – | – | – | 0.051[a] | 0.051[a] | 0.137[a] | -0.013[a] | -0.014[a] | -0.101[a] |
| **Prognosis risks of CKD** | – | – | – | – | – | – | – | – | 0.128[b] | 0.229[b] | 0.163[b] | 0.195[b] | -0.124[b] |
| **hsCRP** | – | – | – | – | – | – | – | – | – | 0.442[b] | 0.318[b] | 0.234[b] | -0.013[b] |
| **IL-6** | – | – | – | – | – | – | – | – | – | – | 0.302[b] | 0.161[b] | -0.018[b] |
| **WBC** | – | – | – | – | – | – | – | – | – | – | – | 0.282[b] | 0.165[b] |
| **HOMA-IR** | – | – | – | – | – | – | – | – | – | – | – | – | 0.152[b] |

**Abbreviation:** HTN, Hypertension; DM, Diabetes Mellitus; MetS, Metabolic Syndrome; CKD, Chronic Kidney Disease; hsCRP, High Sensitive C-Reactive Protein; IL-6, Interleukin-6; WBC, White Blood Cell; HOMA-IR, Homeostasis Model Assessment of Insulin Resistance.

Note: [a] Point-biserial correlation is calculated between a dichotomous variable and a continuous variable.

[b] Spearman rank correlation is calculated between two continuous variables.

[c] Phi coefficient is calculated between two dichotomous variables.
