# Supplementary material for: Independent association between subjective cognitive decline and frailty in the elderly
Source: PLoS One. 2018 Aug 2;13(8):e0201351. doi: 10.1371/journal.pone.0201351 (PMC6072005; doi:10.1371/journal.pone.0201351)
Supplement: S2 Table — (PDF) [file pone.0201351.s002.pdf]

Supporting information: **Independent association between subjective cognitive decline and frailty in the elderly**

S2 Table. Odds ratios of SCD versus normal cognition for the elderly with pre-frailty or frailty (robust as reference category) in unadjusted and adjusted models.

Models	Covariates used	SCD versus normal cognition for pre-frailty OR (95% CI)	*p*-value	SCD versus normal cognition for frailty OR (95% CI)	*p*-value	*p*-value for interaction effects between SCD and added-covariates
1	SCD (unadjusted model)	1.42 (1.16–1.74)	< 0.001	1.91 (1.36–2.69)	< 0.001	–
2	Model 1 plus age	1.47 (1.20–1.80)	< 0.001	2.09 (1.47–2.97)	< 0.001	0.579
3	Model 2 plus gender	1.45 (1.18–1.78)	< 0.001	2.05 (1.44–2.92)	< 0.001	0.083
4	Model 3 plus education level	1.41 (1.15–1.73)	0.001	1.95 (1.36–2.78)	< 0.001	0.111
5	Model 4 plus HTN	1.38 (1.13–1.70)	0.002	1.90 (1.33–2.71)	< 0.001	0.791
6	Model 5 plus stroke	1.38 (1.13–1.70)	0.002	1.90 (1.33–2.72)	< 0.001	0.893
7	Model 6 plus DM	1.36 (1.11–1.68)	0.003	1.83 (1.28–2.63)	0.001	0.482
8	Model 7 plus Mets	1.36 (1.11–1.67)	0.004	1.82 (1.27–2.61)	0.001	0.477
9	Model 8 plus poor nutritional status	1.36 (1.11–1.67)	0.004	1.81 (1.26–2.60)	0.001	0.780
10	Model 9 plus prognosis of risks of CKD	1.36 (1.10–1.67)	0.004	1.78 (1.23–2.56)	0.002	0.957
11	Model 10 plus hsCRP	1.36 (1.10–1.67)	0.004	1.77 (1.23–2.55)	0.002	0.696
12	Model 11 plus IL-6	1.36 (1.10–1.68)	0.004	1.79 (1.24–2.58)	0.002	0.320
13	Model 12 plus WBC	1.36 (1.10–1.67)	0.004	1.78 (1.23–2.57)	0.002	0.883
14	Model 13 plus HOMA-IR	1.35 (1.10–1.67)	0.005	1.77 (1.22–2.55)	0.003	0.766
15	Model 14 plus hemoglobin	1.36 (1.10–1.67)	0.004	1.78 (1.23–2.58)	0.002	0.212

Abbreviation: SCD, Subjective Cognitive Decline; HTN, Hypertension; DM, Diabetes Mellitus; MetS, Metabolic Syndrome; CKD, Chronic Kidney Disease; hsCRP, High Sensitive C-Reactive Protein; IL-6, Interleukin-6; WBC, White Blood Cell; HOMA-IR, Homeostasis Model Assessment of Insulin Resistance.
